# Conceptual and operational definitions of the components of the nursing diagnosis Acute Pain (00132)

**DOI:** 10.1590/1518-8345.2330.2973

**Published:** 2017-12-21

**Authors:** Marisa Dibbern Lopes Correia, Erika Christiane Marocco Duran

**Affiliations:** 1 Doctoral Student, Faculdade de Enfermagem, Universidade Estadual de Campinas, Campinas, SP, Brazil. Adjunct Profesor, Departamento de Medicina e Enfermagem, Universidade Federal de Viçosa, Viçosa, MG, Brazil.; 2 PhD, Professor, Faculdade de Enfermagem, Universidade Estadual de Campinas, Campinas, SP, Brazil.

**Keywords:** Nursing, Acute Pain, Validation Studies, Nursing Diagnosis, Intensive Care Units, Review

## Abstract

**Objective:**

to develop the operational and conceptual definitions of the defining characteristics and related factors of the nursing diagnosis Acute Pain (00132) for nonverbal critically ill patients.

**Method:**

integrative literature review in the databases/libraries: Medical Literature Analysis and Retrieval System Online (MEDLINE via Pubmed), Cochrane Library, The Cumulative Index to Nursing and Allied Health Literature (CINAHL) and Latin American & Caribbean Health Sciences Literature (LILACS).

**Results:**

799 results were found in the literature, of which 80 studies were selected for full text reading and 16 were used in the elaboration of definitions for the 17 defining characteristics and three related factors of the nursing diagnosis. The gray literature, ie, thesis, dissertations, books, guidelines and dictionary was also explored to ensure the robustness needed to clarify the topics not covered by the studies.

**Conclusion:**

the definitions aim to facilitate the identification of the nursing diagnosis for nonverbal critically ill patients and to support future teaching and research on the nursing diagnosis of Acute Pain (00132).

## Introduction

Pain is defined as “an unpleasant sensory and emotional experience associated with actual or potential tissue damage, or described in terms of such damage”^1^, and it can be influenced by multiple factors. It is the most common subjective condition to lead people to see a health professional^2^ ^).^

In the intensive care unit (ICU), pain is a consequence of therapeutic and diagnostic procedures, an inadequate pain relief can have deleterious effects on patients, such as altered respiratory mechanics, increased cardiac demand, muscle spasms, contractions and even muscle rigidity^3^. On the other hand, pain can be difficult to assess in critically ill patients, delaying their treatment. A study conducted through post-discharge interviews with ICU patients found that 63% had had moderate to severe pain during their stay in the ICU and had difficulties to communicate their pain to the health team^4^.

Verbal communication is gold-standard for assessing pain. However, the inability to communicate verbally, present in many patients in intensive care units, does not negate the possibility that they are experiencing pain and need to have treatment^1^.

Due to this problem, statistics for pain prevalence are practically non-existent for nonverbal patients and patients suffering from severe illnesses or with cognitive impairment are often excluded from pain prevalence studies^5^. However, a large study called Thunder II^3^ listed procedures that can cause pain. In adults, the procedures with higher levels of pain were turning in bed, drain removal, wound care, tracheal suctioning with a lower score, and central catheter placement. These are common procedures, constantly performed in ICUs. Therefore, it is fundamental to study, identify and control pain in this environment.

In their daily practice, ICU nurses must have quick decision-making skills based on the observation of physiological and behavioral changes in patients. The direction of the nursing care depends on the nurses’ rapid observation, since complications that affect the recovery of their patients can be prevented^2^.

This rapid assessment is expressed through the identification of the nursing diagnosis (ND). For this, nursing professionals can rely on their own taxonomies, such as the NANDA International classification (NANDA-I)^6^, which provides standardized terms and helps making the best choice of interventions to achieve favorable nursing outcomes, always taking the nurses’ clinical judgment into account^7^. In order to make an ND, the nurse needs to assess the signs and symptoms presented by the patient, which are called defining characteristics (DC), and identify the contributing factors that led to that ND, called related factors (RF). Studying all these elements is essential, revising and evaluating their application in different populations and addressing as many indicators and terms as possible in order to determine accuracy and support their use in diverse populations.

These studies aim to improve the DCs commonly identified for the NDs^8^. Currently, these studies proposed by nurses have sought to study these DCs in specific populations. Therefore, NDs are based on evidence that can be generalized^8^ and used by nursing professionals.

These validation studies provide refinement and improvement of classifications, which favors the exercise of critical thinking and decision-making and improves communication and standardized nursing records^7^.

Therefore, the objective of this study is to develop conceptual and operational definitions of each DC and conceptual definitions of the related factors present in NANDA-I^6^ for the ND Acute Pain (00132). The development of the definitions aims to support nurses in the identification of these characteristics in the population and represents the initial phase of the validation study.

## Method

The methodological process of this integrative review was in agreement with the recommendations of the Preferred Reporting Items for Systematic Reviews and Meta-Analyzes - PRISMA^9^. This process consisted of the following steps: identification of the problem; literature search; evaluation and selection; analysis and presentation of data^10^.

The selected studies were thoroughly read and data were extracted using an instrument validated in Brazil^11^, which included the identification of the study, setting of the study, the characteristics of the journal, the methodological characteristics and an assessment of methodological rigor. After data extraction, an information table was constructed with data from the studies: authors, title, journal, country, language, year of publication, objective, design, population, result and level of evidence. The level of evidence was classified in seven levels^12^: level I, evidence from a systematic review or meta-analysis of all relevant randomized controlled trials or from guidelines based on systematic reviews of randomized controlled trials; level II, evidence obtained from at least one well-designed randomized controlled trial; level III, evidence from well-designed controlled trials without randomization; level IV, evidence from cohort or case-control studies; level V, evidence from systematic reviews of descriptive and qualitative studies; level VI, evidence from a single descriptive or qualitative study; level VII, evidence from the opinion of authorities and/or reports of expert committees.

Between February 8^th^ and 16^th^, 2017, an integrative review was conducted with the literature available at the databases Medical Literature Analysis and Retrieval System Online (MEDLINE via Pubmed), Cochrane Library, The Cumulative Index to Nursing and Allied Health Literature (CINAHL), and in the Latin American & Caribbean Health Sciences Literature (LILACS). These databases were chosen due to their broad coverage in the areas of health and nursing.

Medical Subject Headings Terms, Health Science Descriptors and key words from 17 DCs and 3 RFs were used in the literature search along with term “dor aguda/acute pain” or “dor”, according to the result obtained. For the DCs “expressive behavior” and “narrowed foccus” in the MEDLINE database (via Pubmed) the time criteria was withdrawn, since there were relevant results from the year 2011. The term “dor” was used only in the LILACS database, since many DCs and RFs did not have any result with the term “dor aguda/acute pain”, so this strategy was used to increase the number of results. The inclusion criteria were: results addressing pain in humans, published between the years 2013-2017, in English, Spanish or Portuguese.

**Figure 1:**
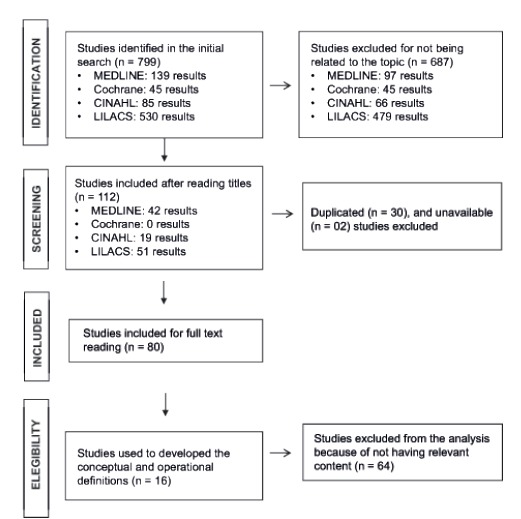
Flow diagram of the review phases

After reading the titles and abstracts, 51 studies were selected for full text reading in Lilacs, 42 in MEDLINE, 19 in CINAHL and none in Cochrane. After the exclusion of the duplicates (n=30) and the unavailable studies (n=2), 80 studies proceed for full text reading. Of these, 16 had relevant content for the development of the conceptual and operational definitions of the DCs and RFs presented in NANDA-I for the ND Acute Pain (00132), according to Figure 1.

As it was not possible to elaborate all the conceptual and operational definitions using only the studies of the systematic review, gray literature was also explored. Thus, a dictionary^13^, two dissertations^14^ ^-^ ^15^, a thesis^16^, two books^17^ ^-^ ^18^ and a guideline^19^ were included, totaling 23 items that supported the development of conceptual and operational definitions of the 17 DCs and three RFs present in NANDA-I for the ND Acute Pain (00132).

## Results

The studies selected supported the development of this study. None of them had level of evidence I or II, due to the characteristics of the subject; however, they provided valuable contributions that supported the development of the definitions.

The journals in which the studies were published are worth highlighting: British Journal of Anaesthesia, Canadian Journal of Emergency Medicine, Escola Anna Nery Revista de Enfermagem, International Journal of Behavioral Medicine, International Journal of Nursing Terminologies and Classifications, Pain Management Nursing (four studies), Revista Colombiana de Psiquiatría, Revista Dor (two studies), Revista Latino-Americana de Enfermagem, Revista de Neuro-psiquiatría e The Journal of Pain (two studies). 

**Figure 2:**
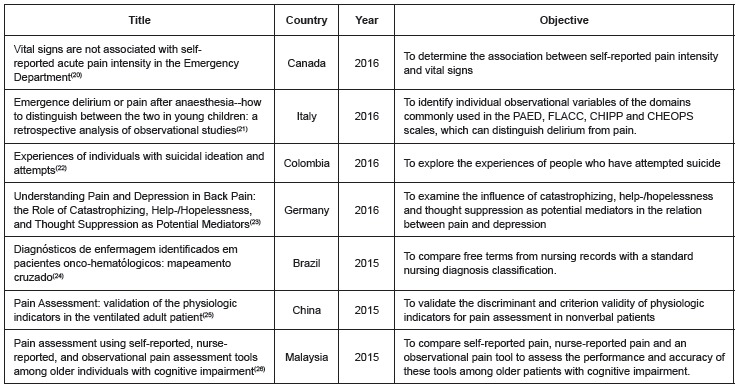
Characterization of the studies published in 2015 and 2016 regarding title, country and objectives

**Figure 3:**
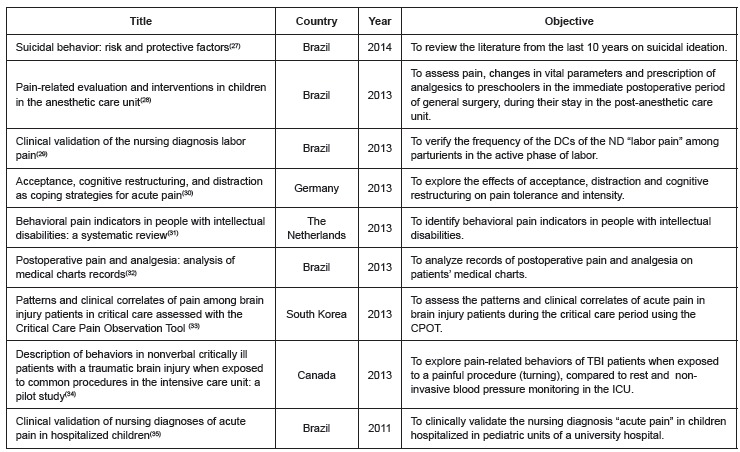
Characterization of the studies published between 2011 and 2014 regarding title, country and objectives

The studies included in the review, classified according to title, country, year and objective, are presented in Figures 2 and 3.

Regarding language, ten studies were in English^20^ ^-^ ^21^ ^,^ ^23^ ^,^ ^25^ ^-^ ^26^ ^,^ ^30^ ^-^ ^31^ ^,^ ^33^ ^-^ ^35^, four in Portuguese^24^ ^,^ ^28^ ^-^ ^29^ ^,^ ^32^ and two in Spanish^22^ ^,^ ^27^. Six studies were conducted in Brazil, two in Germany and Canada and the others in China, Colombia, South Korea, the Netherlands, Italy and Malaysia.

There were two reviews and 14 clinical studies, of which eleven were conducted with adults and three with children. Only two were conducted in critical care units. Six studies excluded the patients who could not communicate, which highlights the need to promote further research for this specific population.

Regarding pain assessment, the verbal report of the patient, without the use of an appropriate instrument^35^, and proxy reports^26^ were used. In addition, several self-report instruments were used, such as the visual numeric scale^20^ ^,^ ^23^ ^,^ ^25^ ^,^ ^28^ ^-^ ^29^, the analog scale ^30^ ^,^ ^32^ and the McGill Pain Questionnaire^29^. Some specific instruments were also used to assess pain in the articles selected: the PAINAD (Pain Assessment in Advanced Dementia)^26^ was used for patients with dementia, the FLACC (Faces, Legs, Activity, Cry, and Consolability)^21^, the CHIPP (Children’s and Infants’ Postoperative Pain)^21^ and the CHEOP (Children’s Hospital of Eastern Ontario Pain)^21^ for children and PBAT (Pain Behavior Assessment Tool)^34^ and CPOT (Critical-Care Pain Observation Tool)^27^ ^,^ ^34^, both observational tools, were used for patients who couldn’t communicate.

Regarding the method, there were descriptive and qualitative studies, systematic reviews of the literature and of patient records, observational studies and controlled studies without randomization.

The evidence levels^12^ varied between level III (evidence from well-designed controlled trials without randomization)^23^, level IV (evidence from cohort or case-control studies)^15^ ^-^ ^16^ ^,^ ^18^ ^,^ ^22^ ^,^ ^27^ ^-^ ^28^, level V (evidence from systematic review of descriptive and qualitative studies)^20^ ^,^ ^23^ ^)^ and level VI (evidence from a single descriptive or qualitative study)^13^ ^-^ ^14^ ^,^ ^17^ ^,^ ^19^ ^,^ ^21^ ^,^ ^25^ ^-^ ^26^.

The operational and conceptual definitions of the DCs for the ND Acute Pain (00132), developed after integrative review of the literature and additions from the gray literature, are presented next:

*Self-report of intensity using standardized pain scale (e.g., Wong-Baker FACES scale, visual analogue scale, numeric rating scale)* - Conceptual definition: It is a subjective evaluation^20^ in which the person in pain^14^ ^)^ makes a verbal report^13^ characterizing pain intensity^32^, that is, the person evaluates and quantifies their level of pain; Operational definition: Verbal report of conscious and minimally verbal person^33^, requested to quantify their pain using standardized scales appropriate for their age group/clinical condition and translated and validated in Brazil.

*Self-report of pain characteristics using standardized pain instrument (e.g., McGill Pain Questionnaire, Brief Pain Inventory)* - Conceptual definition: It is a subjective evaluation^20^ in which the person in pain^14^ ^)^ makes a verbal report^13^ ^)^ characterizing aspects of their pain other than intensity: location^32^, quality^18^ ^,^ ^28^ ^,^ ^30^ ^,^ ^32^ ^-^ ^33^, duration^28^, improvement and worsening factors^32^, factors associated^32^, affective influence^28^; Operational definition: Verbal report of conscious and minimally verbal person^33^, requested to evaluate their pain regarding location, quality, duration, improvement and worsening factors and affective influence, using standardized scales appropriate for their age group/clinical condition and translated and validated in Brazil.

*Distraction Behavior* - Conceptual definition: It is the lack of concentration in the surroundings^13^; little attention to environmental stimuli^30^; lack of attention^14^. It is an adaptation behavior^30^ to a painful event, through which the person remains distracted and away from the real world; Operational definition: Identified through concentrated observation^18^ ^)^ to recognize the individual oblivious to the environment.

*Expressive behavior (e.g., restlessness, crying, vigilance)* - Conceptual definition: Expressive behavior refers to behavioral reactions to stimuli^35^. Some behaviors demonstrated can be indicators of pain, such as: irritability^29^ ^,^ ^35^, anxiety^35^, restlessness^35^/agitation^29^, social isolation^35^, fear^35^, vigilance^29^, sighing^29^, moaning^29^ ^,^ ^35^ and crying^29^ ^,^ ^35^; Operational definition: Identified through concentrated observation^18^ and interaction (active listening and discourse analysis)^18^ to find the indicators irritability, anxiety, restlessness/agitation, social isolation, fear, vigilance, sighing, moaning and crying.

*Protective Behavior* - Conceptual definition: Reaction to external aggression (physical examination, procedures) in an attempt to protect or defend^13^ the area of ​​the body affected by pain; Operational definition: Identified through concentrated observation^18^ to spot the individual flinching the region of the body affected by the pain when it receives stimuli (touch, compression, physical contact).

*Hopelessness* - Conceptual definition: It’s an emotion^23^ related to lack of hope ^(^ ^13^, of trust in something positive^13^ and of meaning of life^24^. It is a negative subjective perception^22^ on pain and a feeling of hopelessness of improving the prospects for the future^27^; Operational definition: Identified through interaction (active listening and discourse analyses)^18^ to recognize negative verbal reports about the future regarding the pain. E.g.: “This pain will never go away”, “This pain will kill me”.

*Diaphoresis* - Conceptual definition: It is the secretion of sweat^13^ and perspiration in abundance^16^ ^,^ ^35^, as an answer from the autonomic nervous system to the stress^35^ caused by pain; Operational definition: Identified through inspection^18^ of evident perspiration on the face, hands, armpits and skin folds^18^. It can be confirmed by using a paper towel on the visually affected site^16^.

*Pupil dilation* - Conceptual definition: Increased pupil diameter^13^, larger than 5 mm^18^, due to the stimulation of the sympathetic nervous system^14^ ^,^ ^16^ caused by the painful event; Operational definition: Identified through inspection^18^ of a pupil size (diameter) larger than 5 mm^18^ using a pupilometer^16^ ^)^ before, during and after the painful event. Record size immediately after elevation of upper eyelids.

*Evidence of pain using standardized pain behavior checklist for those unable to communicate verbally (e.g., Neonatal Infant Pain Scale, Pain Assessment Checklist for Seniors with Limited Ability to Communicate)* - Conceptual definition: It is the use of a scale or classification previously validated to identify the existence of pain in a specific population (neonates, children, adults, older adults with dementia or communication problems, people under mechanical ventilation); Operational definition: Pain assessment through the identification of the presence or absence of the items on the scales. E.g. Behavioural Pain Scale (BPS) for adults in mechanical ventilation, validated in Brazil.

*Facial expression of pain (e.g., eyes lack luster, beaten look, fixed or scattered movement, grimace)* - Conceptual definition: It is the way the face^13^ expresses pain through facial mimics^14^, presenting a facial expression usually different^21^ from when there is no pain; Operational definition: Identified through inspection^18^ of face characteristics (facial mimics) looking for the indicators: grimace^34^, beaten look, tense face^31^, deeper naso-labial furrow^31^, wrinkled forehead^34^, eyes closed tight with wrinkled eyelids^31^ ^,^ ^34^, watery eyes^31^ ^,^ ^34^, raised eyebrows^34^, open lips^14^, clenched teeth^16^.

*Self-focused* - Conceptual definition: Focus is the point to which something converges^13^. Self-focus is a strategy to conserve energy; the individual on pain tries to protect himself, saving energy to go through the period of suffering. This is done through immersion in oneself and self-centering, to concentrate all vital force to cope with the moment of pain; Operational definition: Identified through observation^18^ to recognize an individual who is not very communicative, and may have closed eyes and little contact with others.

*Narrowed focus* - Conceptual definition: Focus is the point to which something converges^13^. An individual with narrowed focus uses it as a self-protection mechanism, preventing that other people or the environment bring more suffering than the pain already endured. This resource is a defense mechanism against external aggressions that could delay recovery or increase suffering; Operational definition: Identified through observation^18^ to recognized an individual with little or no interaction with people or the environment. E.g. Talks little, usually in a quiet voice, only responds to what is necessary, almost monosyllabic, avoids long and detailed dialogue, tries not to move voluntarily or interact with the environment, opts for a peaceful, cozy, dull and quiet environment.

*Protective Behavior* - Conceptual definition: Voluntary or involuntary body movement^13^ revealing an intention to relieve pain of a certain area of ​​the body; Operational definition: Identified through concentrated observation^18^ to perceive the individual touching, holding or compressing a body part affected by pain.

*Appetite change* - Conceptual definition: Change in the desire to eat^13^ (increase, decrease or absence of habitual food intake), regardless of the type of food offered^14^, initiated with (and because of) the occurrence of painful event^14^; Operational definition: Identified through the verbal report of changes in food intake and/or observation of the food intake pattern^16^.

_^*Change in physiological parameter (e.g., blood pressure, heart rate, respiratory rate, oxygen saturation, and end-tidal CO2)*^_ - Conceptual definition: Changes in vital signs caused by the release of catecholamines^25^ as a response to painful stimuli: *increase in blood pressure: blood pressure is the force of blood against the side walls of blood vessels. Systolic blood pressure (SBP) is the maximum pressure perceived during contraction of the left ventricle or systole. The diastolic blood pressure (DBP) corresponds to a period of rest between each contraction. The mean arterial pressure (MAP) is the pressure that forces the blood towards the tissues and it corresponds to the mean of the entire cardiac cycle. This is an arithmetic mean between systolic and diastolic pressures, since diastole lasts longer^18^. Reference values: SBP greater than or equal to 140mmHg and/or DBP greater than or equal to 90mmHg^19^; MAP between 70 and 105 mmHg^17^; *increase in heart rate: number of heart beats in one minute. In the normal adult it varies from 60 to 100 beats per minute^18^; *increase in respiratory rate: number of respiratory cycles in one minute. In normal adults it varies from 10 to 20 breathes per minute^18^; Operational definition: arterial hypertension: SBP values ​​greater than or equal to 140mmHg and/or DBP greater than or equal to 90mmHg; MAP greater than 105 mmHg; heart rate: values ​​greater than 100 beats per minute; respiratory rate: values ​​greater than 20 breathes per minute.

*Positioning to ease pain* - Conceptual definition: Unusual body posture^15^ adopted by the individual in an attempt to avoid or minimize pain^14^; Operational definition: Identified through concentrated observation^18^ of the posture adopted, looking for fetal position, child’s pose or other postures^15^.

*Proxy report of pain behavior/activity change (e.g., family member, caregiver)* - Conceptual definition: Exposition, description or narration^13^ of a caregiver, family member or member from the health team familiar with the behavior of the person, about a change in their behavior^26^ that could indicate the presence of pain; Operational definition: Identified through the verbal report of caregiver, family member or health team member about changes perceived in the behavior of the individual (pain faces, body position, appetite change).

The conceptual definitions of the RFs for the ND acute pain (00132), constructed after an integrative review of the literature and additions from the gray literature, are presented next:

*Biological injury agent (e.g., infection, ischemia, neoplasm)* - Conceptual definition: Something that causes an injury (pathological or traumatic tissue damage)^13^ related to a biological event (alteration in the cellular function of an organ or tissue) or invasion of microorganisms.

*Physical injury agent (e.g., abscess, amputation, burn, cut, heavy lifting, operative procedure, trauma, overtraining)* - Conceptual definition: Something that causes an injury (pathological or traumatic tissue damage)^13^ ^)^ related to a physical event of an accidental or surgical damage on organic tissues.

*Chemical injury agent (e.g., burn, capsaicin, methylene chloride, mustard agent)* - Conceptual Definition: Something that causes an injury (a traumatic tissue change)^13^ caused by a chemical agent (e.g. capsaicin, methylene chloride, mustard agent).

## Discussion

The symptom of pain is a pressing issue among hospitalized patients, since millions of people suffer from pain as a result of trauma, surgery or diseases^36^. Pain management depends on the correct assessment by nurses, who must identify pain among the patients under their care. The objective of this action is to minimize the undesirable effects of inadequate pain management such as reduced quality of life, impaired sleep, delayed recovery, and the risk of developing chronic pain^36^.

This study aims to help nurses in clinical practice to correctly name and identify the signs and symptoms (DCs) presented by the patient and the related factors (RFs) for the correct indication of the ND Acute Pain (00132). Thus, with the indication of an ND, the care plan can be correctly implemented and the patient can receive the best available treatment to alleviate their suffering and prevent complications from inadequate pain management. 

Therefore, a validation study addressing the components of the ND to improve the already existing taxonomies can be essential, since it can give the necessary strength to the Nursing praxis, increasing the visibility of the profession. In addition, studies indicate gaps in the knowledge about pain of the nursing team on critical care units^37^ ^-^ ^39^, which makes this study even more important.

Moreover, since the conceptual and operational definitions clarify and reinforce the items in an ND, this study contributes to the development of specific language, which allows for future discussions on the best available evidence on these components and their applicability to specific populations.

Validation studies provide refinement and improvement of classifications, which favors the exercise of critical thinking and decision-making and improves communication and standardized nursing records^7^.

The use of standardized language favors universality and dissemination of information. Therefore, it also benefits teaching activities, since students can better understand the phenomenon studied.

The present study aimed to elaborate the conceptual and operational definitions of the DCs and the conceptual definitions of the RFs of the ND Acute Pain (00132), addressing nonverbal patients in the ICU. The importance of describing this phenomenon is evident, since it can provide the scientific support for nurses to identify an ND that represents, in fact, the patient’s response^40^. In addition, the knowledge about the conceptual and operational definitions can support nursing teaching and future researches of the phenomenon.

In addition to the frequency of the ND, it is relevant to determine the accuracy of the defining characteristics for specific populations. This knowledge can help understanding how the human response is presented, with its antecedents and consequences, and can also support a quality care^40^. A content validation study developed with nurses from the Czech Republic^41^ found that the DCs positioning to ease pain, observed evidence of pain, verbal report of pain, protective gestures, protective behavior, changes in heart rate, expressive behavior and sleep disorders were considered by the nurse judges as major DCs, that is, the DCs that best defined patients with pain. On the other hand, nurses from Slovakia evaluated in the same study only pointed as major DCs positioning to ease pain, observed evidence of pain, verbal report of pain and protective gestures.

Another clinical validation study^42^ conducted with Brazilian children in a teaching hospital found 13 major DCs: expressive behavior, changes in mental status, verbal report of pain, observed evidence of pain, narrowed focus (or self-focus - this study grouped those into a single DC), protective gestures, positioning to ease pain, sleep disorders, protective behavior, changes in heart rate, changes in muscle tone, changes in respiratory rate and facial mimics.

These different data show the variation between nations and populations, but also show some similarities. Therefore, these data indicate the importance of replicating validation studies for different populations and of evaluating the clinical validity of NDs after the content validation.

And, finally, nursing practice is favored by the uniformity in the evaluation and indication of the diagnosis. This way, the patient also benefits, since the nurse will have consistent evidence to make an ND and determine the next stages of the nursing process^40^.

## Conclusion

Pain is a frequent event in ICU patients and this study considers particularly those who can not communicate. The main characteristic of this population hinders the identification of clinical evidences that lead to the ND Acute Pain.

Despite the scarcity of studies addressing this issue for this population, there are methods to describe and evaluate data that allow studying this phenomenon with the use of procedures that are commonly present in critical care.

This way, the use of proper procedures and the identification of the defining characteristics and related factors of this ND must be in the methodology that will lead to the accuracy of these elements for this population.

For this to occur, the development of the conceptual and operational definitions of the DCs and the conceptual definitions of the RFs of the ND Acute Pain (00132) of the NANDA-I, supported by the literature review and additions from gray literature, completes the first stage of a validation study of the ND. The next stages to be developed in this study are the content analysis and clinical validation of this ND for nonverbal critically ill patients.
